# Evaluation of facial appearance in patients with repaired cleft lip and palate: comparing the assessment of laypeople and healthcare professionals

**DOI:** 10.1186/s40902-019-0189-1

**Published:** 2019-02-06

**Authors:** Samar Alhayek, Mohammed Alsalem, Yazeed Alotaibi, Aamir Omair

**Affiliations:** 1 Preventive Dental Science Department, College of Dentistry, King Saud bin Abdulaziz University for Health Sciences, Riyadh, Saudi Arabia; 2 Dental Department, National Guard Health Affairs, Riyadh, Saudi Arabia; 3 Department of Medical Education, College of Medicine, King Saud bin Abdulaziz University for Health Sciences, Riyadh, Saudi Arabia

**Keywords:** Assessment, Cleft lip palate, Facial appearance, Laypeople

## Abstract

**Background:**

The present study aimed to determine whether laypeople and professionals rate the facial appearance of individuals with repaired complete unilateral or bilateral cleft lip and palate (UCLP, BCLP) similarly based on viewing full facial images.

**Methods:**

The study followed a cross-sectional analytical design where five young patients aged 10 to 14 years, who had completed all stages of their unilateral or bilateral cleft lip and palate treatment (bilateral: three, unilateral: two), were evaluated by two groups. The assessment was done by laypeople and 97 qualified professionals (33 orthodontists, 32 plastic surgeons, and 32 oral and maxillofacial surgeons). Professionals were not involved in any stage of the patients’ treatment.

**Results:**

The facial appearance assessment of the professional groups on different facial aesthetics was significantly lower than that of laypeople, and they had higher perceived need for further treatment. On the other hand, laypeople had higher aesthetic ratings and lower perceived need for further treatment. Differences were also observed between the assessments of the professional groups. Participants who had lower aesthetic assessments of the repair tended to report a higher influence of cleft lip and palate on social activities and professional life.

**Conclusion:**

Differences in perception exist between healthcare professionals and laypeople. The discrepancies between the professional groups could be attributed to different treatment modalities and protocols.

## Background

The human face represents the first recognizable image and identification of a person, and disorders of facial structures have a high impact not only on the anatomy, physiology, and function of the facial region but also on the individual’s acceptance and integration in society [1]. Cleft lip and palate is the most common congenital deformity of the head and neck in Saudi Arabia [2]. Inconsistent treatment and management make it difficult to predict the outcomes of such procedures. Lip and nose surgical correction has been shown to be significantly important for cleft lip and palate patients [3]. The overlap of multiple anatomical structures complicates the repair of cleft lip and palate, which can occur with varying severity. Each patient presents a new challenge to the surgeon attempting to repair the cleft, regardless of whether this patient has a unilateral or bilateral cleft, has a narrow or wide cleft, or is syndromic or non-syndromic [4].

Achieving the surgical goal of the repair should include the creation of an intact and appropriately sized upper lip to compensate for the loss of philtrum height on the cleft side, repair of the underlying muscular structure, and primary repair of nasal deformity [5]. Molsted has reported that all of the surgical methods used to treat cleft lip and palate result in the formation of scar tissue, which to various degrees inhibits growth in the entire maxillary complex [6], and this comprises one of the limitations faced by experts. It was also reported that primary bone grafts do not grow as was originally postulated, but rather, they hinder growth with a significant limitation of maxillary development and a dramatic increase in crossbite malocclusion and pseudoprognathism [7]. Friede and Katsaros reported that under the correct circumstances upon which the functional rehabilitation can be successful [8], patients seem to have concerns about the appearance of cleft-related features [3, 9].

A considerable amount of evidence supports the presence of psychosocial limitations in cleft lip and palate patients. Thompson and Kent pointed out heightened levels of depression and anxiety among those with facial disfigurement [10]. In a study conducted by Berk et al., Chinese adults with cleft lip and palate have been shown to have lower self-esteem than control subjects and siblings. It was also found that social anxiety and avoidance are significantly more in the cleft lip and palate group [11]. Finally, the overall dissatisfaction with facial appearance has been found to be a predictor of depression among subjects with clefts and controls [9]. Perceiving outcomes of cleft lip and palate repair vary between providers who are more aware of the anatomical and technical limitations than laypeople who might have different expectations. Several studies have reported that laypeople and professionals perceive facial aesthetics differently [12–14].

The desire to improve facial aesthetics has been reported to be one of the main reasons people seek treatment by an orthodontist [15], or an oral surgeon [16], including patients with cleft lip and palate. The importance of the clinician’s opinion lies in the fact that it can influence patients’ and parents’ perception of the need for treatment. The clinician’s opinion has been shown to be influenced by gender, type of training, and familiarity with the cleft condition [17, 18]. Cleft lip and palate individuals may be biased when assessing their own facial appearance, as previous related experiences may affect judgment [19], although this is not always the case [20]. The aim of this study was to determine whether laypeople and professionals of different backgrounds rate the facial appearance of repaired cleft lip and palate similarly based on viewing full facial photographs. This attempt hopes to identify major disagreements between the groups that could be used to establish preventive and informative programmes aiming to bridge the gap.

## Methods

This cross-sectional study was approved by the Institutional Review Board of King Abdullah International Medical Research Center (RC17/228/R). The study evaluated the assessment of five young patients aged 10 to 14 years, who had completed all stages of their unilateral or bilateral cleft lip and palate treatment (bilateral: three, unilateral: two). The assessment was done by laypeople, parents of other cleft lip and palate patients, and 97 qualified professionals (33 orthodontists, 32 plastic surgeons, and 32 oral and maxillofacial surgeons). Professionals were not involved in any stage of the patients’ treatment.

The surgical treatment of the patients followed various protocols, but all patients received orthodontic treatment at the Orthodontics Clinics of the National Guard Health Affairs in Riyadh, Saudi Arabia. Patients with syndromes and other congenital anomalies or psychological disorders were excluded from the study. Patients and parents were informed of the study and the first five to agree were included after they signed informed consent. Four photographs (frontal face, right lateral face, three-fourth right face, and smile) were taken from each patient (three females and two males) by one investigator under standardized conditions, and with the same photographic setup [21].

**Fig. 1:**
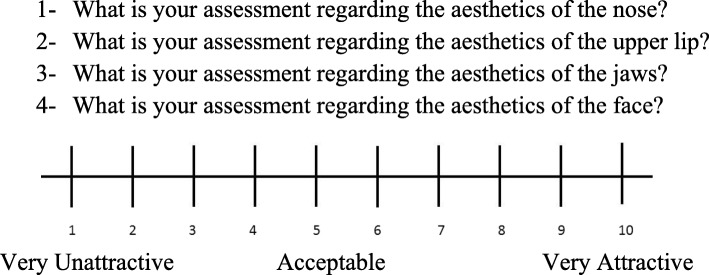
Assessment scale used by raters to evaluate facial aesthetics after repair

Laypeople and professionals evaluated the photos of all subjects under similar conditions using a questionnaire that consisted of four questions for each set of photos, and their answers were recorded on a 10-point visual analogue scale between 1 = very unattractive and 10 = very attractive (Fig. 1). The values were divided into three categories: scores < 4 were considered ‘very unattractive’, scores ≥ 4 and < 7 were considered ‘acceptable’, and scores ≥ 7 were considered ‘very attractive’ [22]. The participants also answered one question about the need for corrective surgery for each of the patients and two questions about the perceived influence of cleft lip and palate (CLP) on social interactions and professional life.

Analysis was done using the Statistical Package for the Social Sciences (SPSS 23 software, Chicago, IL, USA). Statistical analyses were performed on the ratings of laypeople and professional groups. To test for the differences between laypeople, orthodontists, oral and maxillofacial surgeons, and plastic surgeons, data was analysed with one-way analysis of variance (ANOVA). The mean and standard deviation (SD) of all the groups’ ratings were calculated. Independent *t* tests were performed to evaluate the different ratings of the photographs by the professionals and laypeople. In order to test for the relationship between the aesthetic assessment and perceived influence of CLP on social interactions and professional life, Pearson correlation coefficient test was applied. The level of significance was set at .05.

## Results

Ninety-seven healthcare professionals and 100 laypeople participated in the study, of which 83 (42.1%) were females, and 114 (57.8%) were males. Out of the professional group, 33 (34%) were orthodontists, 32 (33%) were oral and maxillofacial surgeons, and 32 (33%) were plastic surgeons. The response rate for orthodontists was 84%, plastic surgeons 79%, oral and maxillofacial surgeons 76%, and laypeople 92%. The mean age for the participants was 35.2 ± 7 years. The mean age and gender distributions for each group are given in Table 1.

**Table 1:** Mean age and gender distribution of the rating panels

Rater group	*n*	Mean age (SD)	Female/male
Orthodontists	33	36.6 (4.6)	14/19
Plastic surgeons	32	38.8 (5.6)	12/20
Oral and maxillofacial surgeons	32	37.8 (5.7)	10/22
Laypeople	100	32.6 (7.5)	47/53

In the attractiveness ratings, the professionals rated the appearance of treated cleft individuals significantly lower in all components of the face [nose (4 ± 1.4), upper lip (4.7 ± 1.4), jaws (4.7 ± 1.2), and face (4.6 ± 1.2)] than the ratings of laypeople [nose (5.1 ± 1.7), upper lip (5.3 ± 1.8), jaws (5.5 ± 1.4), and face (6.4 ± 1)], *P* < .01. Table 2 shows the mean scores of the aesthetic evaluations for all groups.

**Table 2:** Mean assessment scores of the rating panels towards the aesthetics of the nose, upper lip, jaws, and face

Feature	Orthodontists	Plastic surgeons	Oral and maxillofacial surgeons	Laypeople	*P* value
	Mean ± SD	Mean ± SD	Mean ± SD	Mean ± SD	
Nose	4.9 ± 1.3	3.7 ± 1.3	3.6 ± 1.3	5.1 ± 1.7	*P* < .001
Upper lip	5 ± 1.2	4.3 ± 1.5	4.5 ± 1.3	5.3 ± 1.8	*P* < .001
Jaws	5.3 ± 1.1	4.6 ± 1.2	4.1 ± 1	5.5 ± 1.4	*P* < .001
Face	5.2 ± 1	4 ± 1.2	4.7 ± 1.2	6.4 ± 1	*P* < .001

Male participants perceived treatment outcomes as less attractive than what female participants had perceived. However, this finding was not statistically significant (*P =* 0.1). Regarding the perceived influence of CLP on social interactions and professional life, professionals had a perception of higher effect on social interactions (*P =* 0.001) and professional life (*P =* .002) than what laypeople had perceived. Table 3 shows the perceived influence of CLP on social interactions and professional life by laypeople and healthcare professionals.

**Table 3:** Perceived influence of CLP on social interactions and professional life among laypeople and healthcare professionals

Category	Laypeople (*n* = 100)	Professionals (*n* = 97)	*P* value
	Mean ± SD	Mean ± SD	
Effect on social interactions	6.2 ± 2.7	8.4 ± 2.3	0.001
Effect on professional life	6.6 ± 3.2	7.9 ± 2.5	0.002

Pearson correlation revealed a negative relationship between mean facial aesthetic assessment and the perceived influence of CLP on social interactions *r* = − .53 and professional life *r* = − .5 (*P* < .001). In their perception of the need for corrective surgery, plastic surgeons had the highest mean among professionals with a mean of 4.4 ± 1.0, followed by orthodontists with a mean of 3.8 ± 1.4 and finally oral and maxillofacial surgeons with a mean of 3.4 ± 1.8, *P* = .03. In their evaluation of the success of surgical repair, plastic surgeons had the lowest mean of 4 ± 1.3, followed by oral and maxillofacial surgeons with a mean of 4.7 ± 1.2, orthodontists with a mean of 5.7 ± 1, and finally laypeople with a mean of 6.3 ± 1.2, *P* < .001. Table 4 shows the perception of professionals and laypeople towards the success of repair and the need for corrective surgery.

**Table 4:** Perception of participants towards success of repair and the need for corrective surgery

Feature	Orthodontists	Plastic surgeons	Oral and maxillofacial surgeons	Laypeople	*P* value
	Mean ± SD	Mean ± SD	Mean ± SD	Mean ± SD	
Success of repair	5.7 ± 1	4 ± 1.3	4.7 ± 1.2	6.3 ± 1.2	0.001
Need for corrective surgery	3.8 ± 2.4	4.4 ± 1	3.4 ± 1.8	2.5 ± 1.1	0.03

## Discussion

The aim of this study was to evaluate the differences in the assessment of surgical aesthetic facial outcomes of treated cleft individuals by raters of variable backgrounds. Cleft lip and palate patients undergo extensive surgical procedures from birth to adolescence in order to restore function and aesthetics. However, these surgical procedures may result in substantial scarring and disfigurement. Several studies have compared subjective assessments of treatment outcomes between professionals and laypeople [14, 21, 22]. But limited literature exists when it comes to comparing the ratings of treatment outcomes between laypeople and professionals of different backgrounds, including orthodontics and dentofaical orthopaedics, oral and maxillofacial surgery, and plastic surgery.

In the present study, professionals rated treatment outcomes significantly lower than laypeople rated outcomes. This can be attributed to the fact that professionals are more aware of the surgical techniques and gold-standard surgical procedures, which makes them less tolerant of undesirable aesthetic results. This is in contrast with previous studies where there were no differences between the ratings of professionals and laypeople [3, 23, 24]. The different findings could be attributed to the dissimilarity of the rating panels. In the other studies, the rating panels mainly comprised surgeons and laypeople, where the raters in the present study included orthodontists, oral and maxillofacial surgeons, and plastic surgeons, and the lay raters included parents of the cleft-affected individuals. The difference in the panel groups may have affected the aesthetic ratings. However, the rating groups of the present study may produce more representative aesthetic ratings as it accounts for the different professionals of the cleft team who are the most involved group during the course of treatment for these patients, and in fact influence the type and course of therapy. Further studies with a greater number of raters from various cleft team professionals are warranted in order to test the validity of our findings.

Male participants perceived treatment outcomes as significantly less attractive than female participants perceived. Sinko et al. studied different gender perceptions of cleft-affected individuals [3]. They found that female patients with a cleft rated their own facial appearance significantly less than their male counterparts. This could be attributed to the effect of mass media and societal norms in prioritizing females’ physical attractiveness. However, in the present study, male and female raters were not cleft-affected and did not rate their own facial appearance. Instead, they rated other cleft individuals. Limited literature exists on the gender differences in aesthetic perceptions, and further investigations are required.

As for the need for corrective surgery, professionals perceived a greater need for corrective surgery than laypeople. This could be linked to the low treatment expectations of the lay raters and the low perceived influence of cleft lip and palate on social interactions and professional life, thus reflecting a good social acceptance of CLP patient by the general population. Out of the professional panels, plastic surgeons deemed more need for further corrective surgery. This finding is in agreement with Foo et al. [25], who studied the differences between surgical professionals (plastic surgeons) and non-surgical professionals (orthodontist, dentist, and psychologist). Plastic surgeons also had the lowest mean in their assessment of the success of the surgery. This could be a result of the increased treatment options of nose correction and scar remodelling in the field of plastic surgery.

The negative correlation found in the present study between low aesthetic assessments and increased perceived effect of CLP on social interactions and professional life may be associated with the consistent research findings in social sciences that clearly link appearance with social stereotyping and expectations [26, 27].

## Conclusions

Differences in perception exist between professionals who are part of the cleft treatment team and laypeople. Professionals were less satisfied with surgical aesthetic treatment outcomes, while laypeople were more satisfied with the cleft lip and palate repair and did not perceive a high need for corrective surgery as the professional groups did. This discrepancy between the two groups lays the responsibility for the healthcare professionals to offer their patients the best possible treatments, knowing that they could achieve better results by informing them about all treatment options and limitations.

## Abbreviations

BCLP: Bilateral cleft lip and palate
CLP: Cleft lip and palate
UCLP: Unilateral cleft lip and palate
